# Effect of intra-ovarian platelet rich plasma in women with poor ovarian response

**DOI:** 10.22088/cjim.14.3.485

**Published:** 2023

**Authors:** Fatemeh Davari tanha, Shohreh Salimi Setudeh, Mahbod Ebrahimi, Elham Feizabad, Zahra Khalaj Sereshki, Firoozeh Akbari Asbagh, Hamideh Pakniat, Azadeh khalili, Zahra Kaveh, Sara Saeedi

**Affiliations:** 1 Department of Infertility, Yas Hospital, Tehran University of Medical Sciences, Tehran, Iran; 2 Department of Obstetrics and Gynecology, Faculty of Medicine, Qazvin University of Medical Sciences, Qazvin, Iran; 3 Student Research Committee, Tehran University of Medical Sciences, Tehran, Iran

**Keywords:** Ovarian reserve, Assisted reproductive techniques, Pregnancy outcome, Anti-Mullerian hormone, Oocyte quality.

## Abstract

**Background:**

Poor ovarian responder (POR) women, whose ovarian response to gonadotropin stimulation has decreased, are at higher risk of unsuccessful in-vitro fertilization (IVF). Therefore, this study designed to evaluate the effect of intra-ovarian platelet rich plasma (PRP) on POR women.

**Methods:**

This single-arm trial research was done on 20 POR women referred to the IVF Unit, university-based hospital, Tehran, Iran between October 2020 and September 2021. For all participants, autologous PRP was injected into each ovary by transvaginal ultrasound guidance under spinal anesthesia between days 12 and 14 of the menstrual cycle. After 12 weeks of PRP injection, embryo transfers were carried out following our routine IVF department protocol. The study outcomes were the number of mature oocytes, and pregnancy rates.

**Results:**

The average age of the participants was 41.80±1.82 yr. The average infertility duration was 9.70±1.89 yrs., with 80% primary infertility type. After PRP injection, follicle-stimulating hormone levels dropped about 1% (P=0.499), anti-Mullerian hormone levels were on average 4.5% higher (P=0.356), and estradiol levels raised by 1.2% (P=0.681). The average number of oocytes and their quality increased after PRP injection, while these changes were not significant (p-value>0.05). Chemical pregnancy was detected in 3 (15%) women and clinical pregnancy was detected only in one person.

**Conclusion:**

This study revealed that PRP injection into ovaries of POR women is safe and had a tendency to improve ovarian reserve markers and serum levels of AMH, estradiol, number and quality of oocytes.

About 15% of couples are affected by infertility all over the world. Although, assistance-reproductive technology (ART) such as in-vitro fertilization (IVF) helps to resolve this problem in some cases, yet unsuccessful IVF is common in poor ovarian responder (POR) women (1). POR women, whose ovarian response to gonadotropin stimulation has decreased, are at higher risk for ART failure and a large number of them are forced to use donated eggs, which will lead to related cultural and economic problems (2, 3). Recently, the beneficial influence of platelet-rich plasma (PRP) in ovarian tissue regeneration has been one of the most interesting and controversial topics in this regard; because of its confirmed effects in many medical fields such as skin diseases, dentistry, orthopedics, and sports medicine, as well as obstetrics and gynecology indications (4-7). Hence, it was considered that PRP might help ovarian regeneration in POR women because of its role in angiogenesis, follicular vascularization, and activated platelet-derived growth factor (8, 9). 

The strategy of acquiring PRP is non-expensive and low invasive. It consists of a high concentration of platelets found in plasma originating from peripherally collected blood, which carries a large type of proteins, hormones, cytokines, and triggers cell proliferation, growth, and differentiation (10,11). Although former studies showed encouraging results about beneficial influence of PRP in infertile women with improper endometrium thickness (4), recurrent unsuccessful implantation (5), insufficient responses to ovarian stimulation (6), delaying follicle atresia, oocyte degeneration, and ovarian tissue regeneration (7) these studies are few, low sample size and controversial. Therefore, this study was designed to evaluate the effect of intra-ovarian PRP on POR women.

## Methods

This single-arm trial was done on 20 women referred to the IVF Unit, university-based hospital, Tehran, Iran between October 2020 and September 2021. The Tehran University of Medical Sciences Ethics Committee (IR.TUMS.MEDICINE.REC.1399.257) approved the study and the study protocol was registered in Iranian randomized clinical trial registration (IRCT20091012002576N19). The participants signed the informed consent. Inclusion criteria included POR infertile women based on Bologna criteria (12) which are defined as advanced age (≥40 years), history of cycle cancelation, equal or less than 3 oocytes after conventional stimulation protocol, antral follicle count (AFC) <5–7 follicles or anti-Mullerian hormone (AMH) <0.5–1.1 ng/ml, as well as not no infertility causes in her partner. Women with ovarian failure because of gonadal dysgenesis/chromosomal abnormalities, history of major pelvic or abdominal surgeries or pain, anticoagulant or psychotropic drug use, psychiatric diseases, immunoglobulin A deficiency, and cancer were excluded.

For PRP preparation, the venous blood sample of the woman was taken. Then, the blood sample was centrifuged at 1200 rpm for 10 minutes. The upper two-thirds and the middle coat layer were separated from the precipitated red blood cells and centrifuged again at 3300 rpm for 6 minutes. The upper two-thirds of the resulting plasma was then separated and the remaining plasma and precipitate were homogeneous. With this method, about 6 ccs of concentrated platelets with a concentration of 900,000 per microliter were obtained, which can be stored at room temperature for 0-4 hours. After preparation of autologous PRP in the IVF laboratory, it was injected into each ovary by transvaginal ultrasound (TVS) guidance under spinal anesthesia by one expert infertility fellowship and using needle number 17 of ovarian puncture during days 12 and 14 of the menstrual cycle. 

About 12 weeks after the PRP injection, the ART was done as follows. On the second day of expected menstruation, the women were evaluated about endometrial thickness (ET) and AFC with TVS (4.5-7 MHz probe, Sono line G-40, produced with Siemens, Germany). If ET and AFC were proper, 300 micrograms of gonadotropin (CinnalF, CinnaGen Company, Iran) was prescribed. Serial TVS examinations were used to assess follicular maturation. Whenever follicle(s) ≥10-12 mm in average diameter was detected, human menopausal gonadotropin (hMG) (Pooyesh Daru, Iran) and Cerotide (Cetrorelix, SERPERO Company, Swiss) were added. Gonadotropin hormone-releasing hormone (GnRH) antagonist was continued until a mature oocyte by the diameter of 18 mm was observed at TVS, then 250 micrograms recombinant human chorionic gonadotropin (hCG) (Ovitrelle, Merck Serono, Italy) was injected. After 36 hours, a TVS-guided oocyte retrieval was performed under spinal anesthesia. The embryos were transferred on days 3-5 after the procedure. Ampule progesterone (Iran Hormone Company, Iran) was used at 100 mg daily for luteal phase support.

The following data were recorded for all study patients: age, marriage duration, infertility duration and type, body mass index (BMI), as well as follicle-stimulating hormone (FSH), estradiol, luteinizing hormone (LH), AMH, and AFC concentrations before and after PRP injection. The study outcomes were the number of mature oocytes, chemical pregnancy (two weeks after fetal transfer, laboratory positive beta-hCG test) rate, and clinical pregnancy (six weeks after embryo transfer, ultrasound gestational sac by transvaginal sonography) rate. All the data were analyzed through Statistical Package for the Social Sciences (SPSS) Version 24.0. A p-value< 0.05 was considered statistically significant. Independent samples t-test and non-parametric Mann–Whitney U-test was used to evaluate mean differences. A chi-square test and Fisher's exact test were applied to detect the differences in proportion.

## Results

In this study, 32 infertile women were assessed for eligibility; of them, 12 women had been excluded, six due to severe azoospermia in their partners, three due to leiomyoma, and three due to decline to participate. Finally, the data of 20 women were analyzed. The average age of the women was 41.80±1.82 years. The average infertility duration was 9.70±1.89 years, and 80% had primary infertility. The other demographic characteristics are shown in table 1. Women who were treated with PRP had non-significant alternation in biochemical ovarian reserve markers (table 2). After PRP injection, FSH levels dropped about 1% (P=0.499), AMH levels were on average 4.5% higher (P=0.356), and estradiol levels raised by 1.2% (P =0.681). Also, the average number of oocytes and their quality increased after PRP injection, while these changes were not significant (table 3). Of the 20 participants, chemical pregnancy was detected in 3 (15%) women and clinical pregnancy was detected only in one person.

**Table 1:** The demographic characteristics of participants

**Variables**	**Mean±SD**	**Minimum**	**Maximum**
**Female age**	41.80±1.82	40	46
**Body mass index**	25.85±3.16	21.23	32.87
**Infertility Duration**	9.70±1.89	7	12
**Endometrial thickness**	8.00±1.25	5	9
**Follicle stimulating hormone**	13.65±1.14	11.0	16.0
**Anti-Mullerian hormone**	0.44±0.18	0.18	0.80
**Estradiol**	810.30±500.74	305	2516

**Table 2:** Biochemical ovarian reserve marker changing

**Variable**	**PRP injection**		**P-value**
	**Before**	**After**	
**Follicle stimulating hormone**	13.65	13.47	0.499
**Anti-Mullerian hormone**	0.44	0.47	0.356
**Estradiol**	810.30	820.50	0.681

PRP: platelet rich plasma

**Table 3.** Frequency of oocyte, embryo quality and pregnancy rate

**Variables**			**Mean±SD**	**P-value**
**Oocyte Number**		**pre-PRP**	4.05±1.14	0.661
		**post-PRP**	4.25±1.16	
**Quality of the oocytes**	**M1**	**pre-PRP**	1.05±0.94	0.089
		**post-PRP**	1.40±0.75	
	**M2**	**pre-PRP**	1.85±1.03	0.334
		**post-PRP**	1.60±0.88	
	**GV**	**pre-PRP**	1.15±1.3	0.811
		**post-PRP**	1.30±1.26	
**Post-PRP Embryo number**			2.75±0.96	
**Post-PRP ** **Quality of the embryos**		**A**	0.40±0.68	
		**B**	1.20±0.69	
		**C**	1.15±0.813	

PRP: platelet rich plasma

## Discussion

This study showed single-dose autologous intra-ovarian PRP injections of POR women result to decrease FSH, and increase AMH, estradiol levels, number of oocytes and their quality, although these changes were not significant and did not improve the pregnancy outcomes. Our study finding was in line with Stojkovska et al.'s (13) study that showed the pregnancy and lives birth rate in POR women after PRP injection did not differ significantly compared to the non-PRP group, although a tendency for improvement in ovarian reserve indices was detected after PRP injection. The possible causes for this non-significant difference might be due to the low sample size studies, low dose of PRP, and short duration follow-up.

In contrast, in Cakiroglu et al.’s study (11), following intra-ovarian autologous PRP injection in women with primary ovarian insufficiency, antral follicle improvement and spontaneously conceived was detected in 70% and 7.4% of them, respectively. Therefore, they concluded that PRP injections, as a simple approach, result in outcome improvement. In comparison to that study, our patients were older, and the quality of oocytes was less. Conversely with our findings, Melo et al.'s (14) study showed a higher FSH, AMH, and AFC levels, and pregnancy rates after PRP, the possible reason for these differences are the applied dose of PRP, which has been prescribed in three consecutive menstrual cycles in Melo et al.'s study.

The mechanism of PRP in ovarian activity resurgence attributes to increases in concentrations of PDGF, TGF-β, IGF-1/2, VEGF, and EGF which result in increasing the number and maturation of preantral follicles, as well as rising in serum level of AMH in infertile women older than 40 years with premature ovarian insufficiency (15,16). The prevalence of POR women due to cigarette smoking, inappropriate diet, and chemo and radiotherapy is increasing, and a huge number of these women are willing to have their own children without using donated oocytes (17-19). 

PRP injection into the ovaries of POR women seems safe and had a tendency to improve ovarian reserve markers and serum levels of AMH, estradiol, and the number of oocytes and their quality. Hence, it should be recommended to these women with the aim of ovarian reserve improvement, especially in younger ones. Although further trials and prospective studies are needed to evaluate PRP intervention in different subjects with different doses. Despite the strong points of this study, there were some limitations such as less sample size with no control group and randomization. 
